# A novel pre-contoured V-shaped rod in one-level pedicle subtraction osteotomy for the treatment of rigid lumbar kyphosis caused by ankylosing spondylitis: technical note and case series

**DOI:** 10.1186/s12891-025-08872-w

**Published:** 2025-07-04

**Authors:** Hongtao Ding, Cheng Zeng, Andrew Y. Xu, Audrey Y. Su, Jeffrey J. Yeung, Xin Chen, Huadong Wang, Yanbin Zhang, Kai Yan, Yonggang Xing, Da He, Bin Xiao

**Affiliations:** 1 Department of Spine Surgery, Beijing Jishuitan Hospital, Capital Medical University, Beijing, China; 2 Department of Orthopaedic Surgery, Warren Alpert Medical School, Brown University, Providence, RI USA

**Keywords:** Ankylosing spondylitis, Spinal kyphosis deformity, Pedicle subtraction osteotomy, One-level osteotomy, Sagittal alignment

## Abstract

**Purpose:**

To introduce and evaluate the feasibility of one-level pedicle subtraction osteotomy (PSO) combined with pre-contoured V-shaped rods in the treatment of spinal kyphosis deformity caused by ankylosing spondylitis (AS).

**Methods:**

Five patients from ages 33 to 47 years old with progressive spinal kyphosis caused by AS underwent one-level PSO, combined with pre-contoured V-shaped rods. Further technical modifications included a PEEK cage, enhanced positioning device, and enlarged osteotomy area. Preliminary short-term clinical and radiographic outcomes were assessed.

**Results:**

The mean operation duration was 306.0 min with an average estimated blood loss of 765.0 ml. No instrument-related or neurological complications occurred during hospitalization. On average, general kyphosis (GK) was corrected from 68.2° to 22.0° and lumbar lordosis (LL) was restored from − 5.4° to 37.6°. The correction angle ranged from 39° to 56° and the osteotomy vertebrae angle was performed between 34° and 51° based on the surgical necessity, averaging 40.8°. Finally, the mean sagittal vertical angle (SVA) was corrected from 231.8 mm to 89.0 mm and the mean chin-brow vertical angle (CBVA) was reduced from 42.6° to 13.8°.

**Conclusion:**

One-level PSO with pre-contoured V-shaped rods at middle lumbar spine is a novel treatment option for the correction of AS-induced lumbar kyphosis and restoration of lumbar lordosis. This alternative technique obtains satisfactory radiographic and clinical outcomes for AS patients without necessitating additional surgery or elevating the risk of complications such as sagittal translation.

## Introduction

Ankylosing spondylitis (AS) is a chronic, rheumatic disease characterized by progressive inflammation of the sacroiliac joints and axial skeleton. Up to 50% of AS patients present with hyperkyphosis, resulting in back pain, abnormal gait, and other coronal and sagittal imbalances which require surgical correction [1, 2]. As follows, restoring sagittal alignment and correcting spinal deformities are the main goals of AS treatments, with the intention of improving quality of life and reducing AS-related complications [3, 4]. One such treatment is spinal osteotomy, which has largely resulted in improved clinical and radiological outcomes, as measured by chin-brow vertical angle (CBVA) and other spinopelvic parameters [1, 5].

Pedicle subtraction osteotomy (PSO) is a widely utilized procedure which corrects rigid kyphotic deformities secondary to AS. One-level PSO is usually appropriate in the context of mild to moderate kyphosis, while two-level PSO provides a higher correction efficacy for severe spinal curvature, though its longer operation duration, greater blood loss, and more extensive surgical trauma result in a higher complication rate versus its one-level counterpart [6, 7]. Moreover, the success rates of traditional kyphosis correction methods used to supplement PSO, such as cantilever bending, compression, and rod angulation, are largely dependent on patient bone quality and limited by the extent of rod angulation achieved during surgery [8]. In addition, complications such as neurological deficits and sagittal translation continue to pose significant challenges during patient recovery [9].

In this study, to improve surgical outcomes, reduce the impact of osteoporosis on procedural efficacy, and decrease the risk of neurological deficits after one-level PSO in the treatment of severe lumbar kyphosis secondary to AS, we modified the surgical process and redesigned the relevant instrumentation. Our surgical strategy comprised three main components: (1) standard PSO with transverse placement of a proper PEEK cage to sustain the anterior column, enabling surgeons to restore the sagittal curve while reducing the risk of dura buckling and sagittal translation; (2) pre-contoured V-shaped (PV) titanium rod to facilitate restoration of lumbar lordotic curvature; (3) an enhanced positioning device to facilitate postural reduction and osteotomy closure. This study aimed to evaluate the feasibility and preliminary outcomes of one-level PSO with PV rods in the treatment of rigid lumbar kyphosis caused by AS.

## Methods

### Selected patients

Five male patients (average 41.0 years) presenting with AS-induced kyphosis were selected for one-level PSO at L3 with PV rod fixation. Surgical indication was a rigid thoracolumbar kyphosis posing significant hindrance to daily activity, such as lying flat on one’s back and walking upright, and related symptoms such as “round-back” which significantly diminished one’s self-esteem. All surgeries were performed by the same team at a single center.

## Radiographical measurements and surgical planning

Five AS patients with kyphosis underwent one-level PSO with PV rods. Preoperative radiographic evaluations were obtained, including standing whole-spine radiographs, computed tomography (CT) with 3-dimensional reconstructions, and magnetic resonance imaging (MRI) if possible. Preoperative photos of the patients standing, sitting, and lying down were collected for surgical planning.

Considering the clinical and radiographic factors mentioned above, a personalized surgical plan was tailored to each patient with the primary goals of minimizing kyphotic deformity, restoring sagittal alignment, achieving a satisfactory CBVA, and normalizing the sagittal vertical axis (SVA). Target vertebral level and expected degree of osteotomy were confirmed according to the previously established hilus pulmonis method [10, 11].

## Surgical technique

**Fig. 1:**
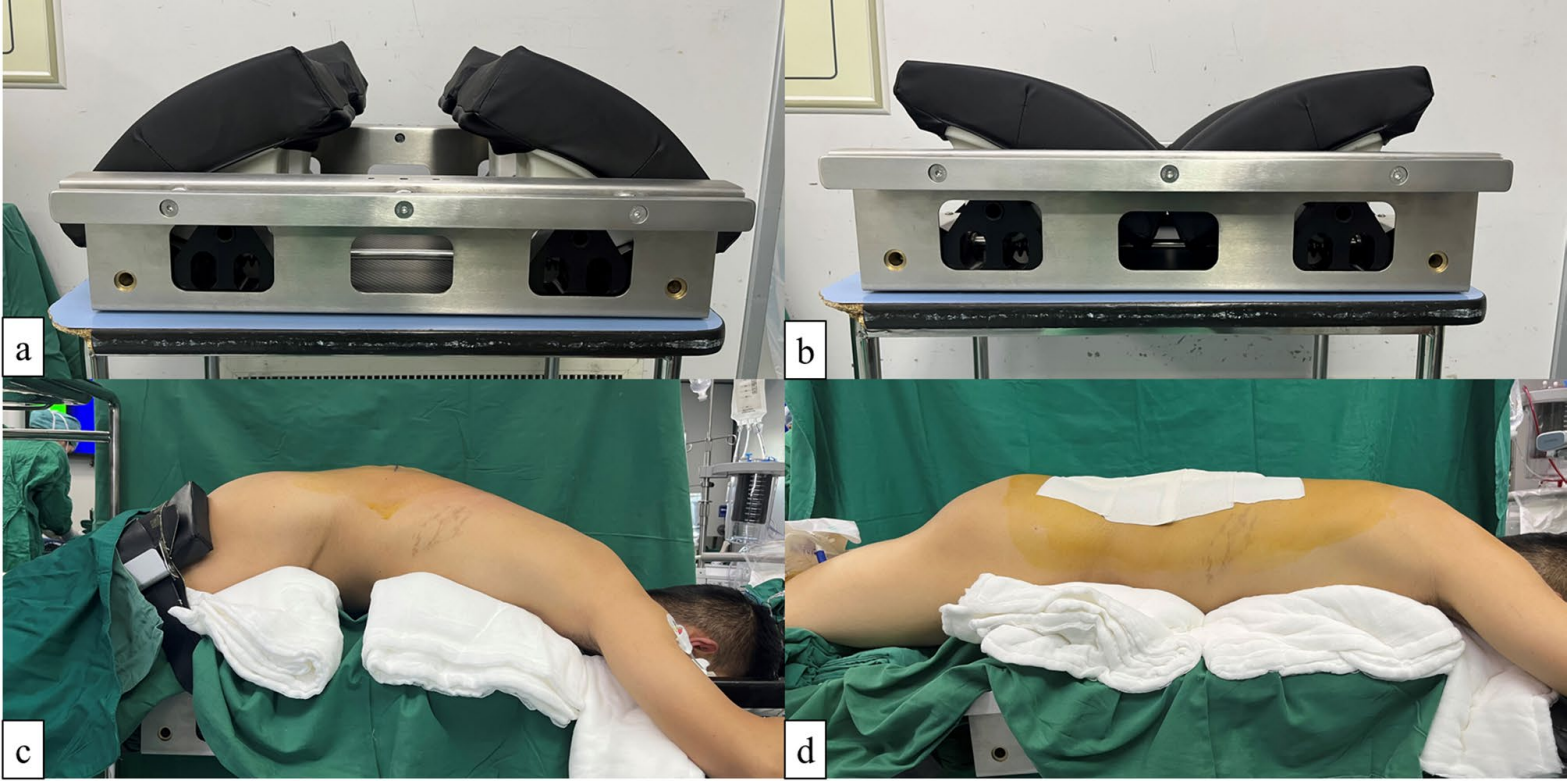
Enhanced positioning device and patient. (**a**), (**b**) enhanced positioning device before (**a**) and after (**b**) postural reduction; (**c**), (**d**) patient position with positioning device under abdomen before (**c**) and after (**d**) postural reduction

Following induction of general anesthesia, each patient was placed in the prone position to avoid potential injury to the cervical spine or other apparatuses. General intravenous anesthesia was administered, and patients were laid upon the enhanced positioning device with the target vertebrae centered (Fig. 1), which facilitated intraoperative posterior column closure. To monitor neurological function, transcranial electrical motor evoked potentials (TCeMEPs) and somatosensory evoked potentials (SSEPs) were watched closely throughout surgery.

A standard posterior midline incision was made, and sub-periosteal exposure of the laminar and lateral masses of the planned region was performed. Pedicle reduction screws were implanted into the planned spinal segments under freehand or navigational guidance, which were at least three levels proximal and two levels distal to the osteotomy site at the L3 vertebrae, to maximize SVA correction following previously-established methods [5, 12].

**Fig. 2:**
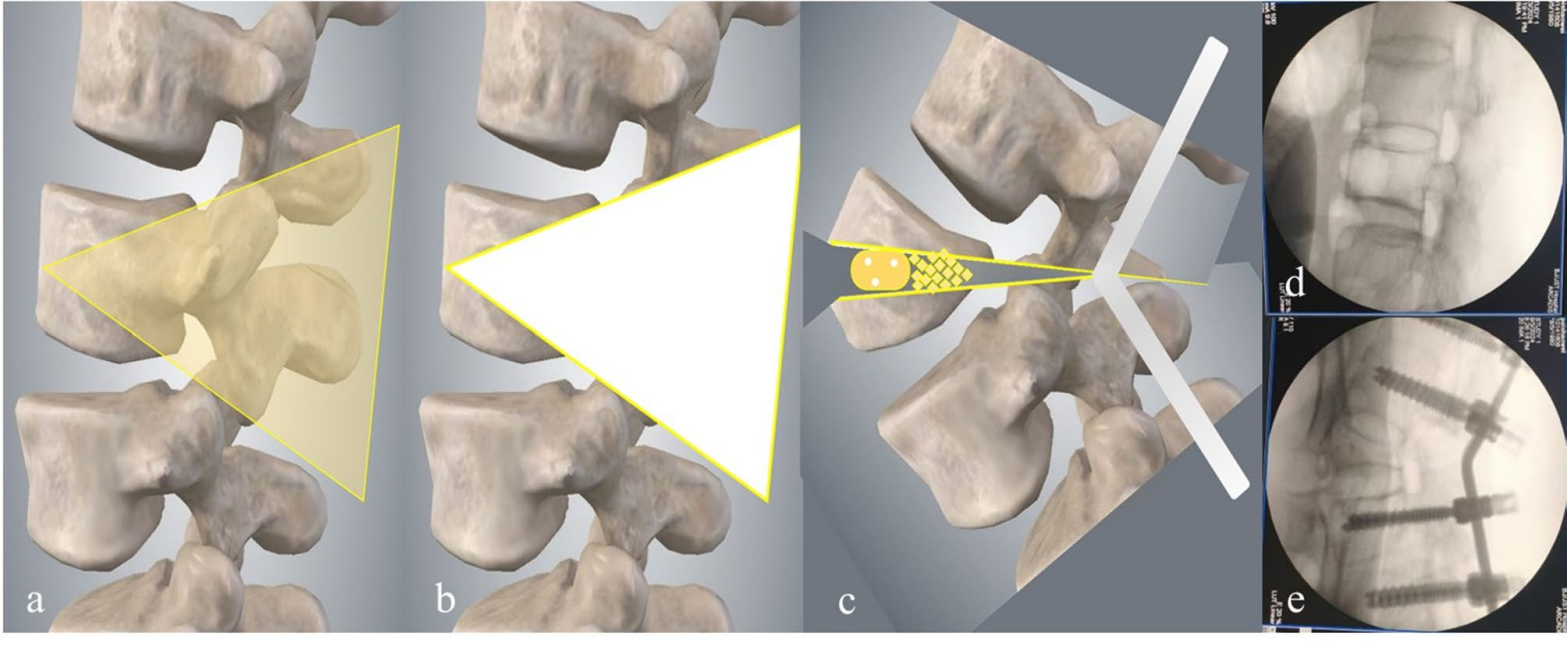
Steps of pedicle subtraction osteotomy (PSO). (**a**) osteotomy area with V-shaped element; (**b**) removal of aforementioned osteotomy elements; (**c**) PEEK cage and autogenous bone graft implanted to restore lumbar lordosis; (**d**) fluoroscopy before osteotomy at the intended segment; (**e**) fluoroscopy after osteotomy and PV rod fixation

Three-column PSO was performed. The articular, spinous, and transverse processes were removed, alongside the pedicles, lower lamina of adjacent cranial vertebrae, and upper lamina of adjacent caudal vertebrae. The extent of bone removal was tailored to ensure adequate space for the neural structures, preventing compression and minimizing the risk of dural buckling during osteotomy closure. An ultrasonic scalpel was used to perform a V-shaped osteotomy (Fig. 2). A temporary rod, PEEK cage, and autograft were implanted.

**Fig. 3:**
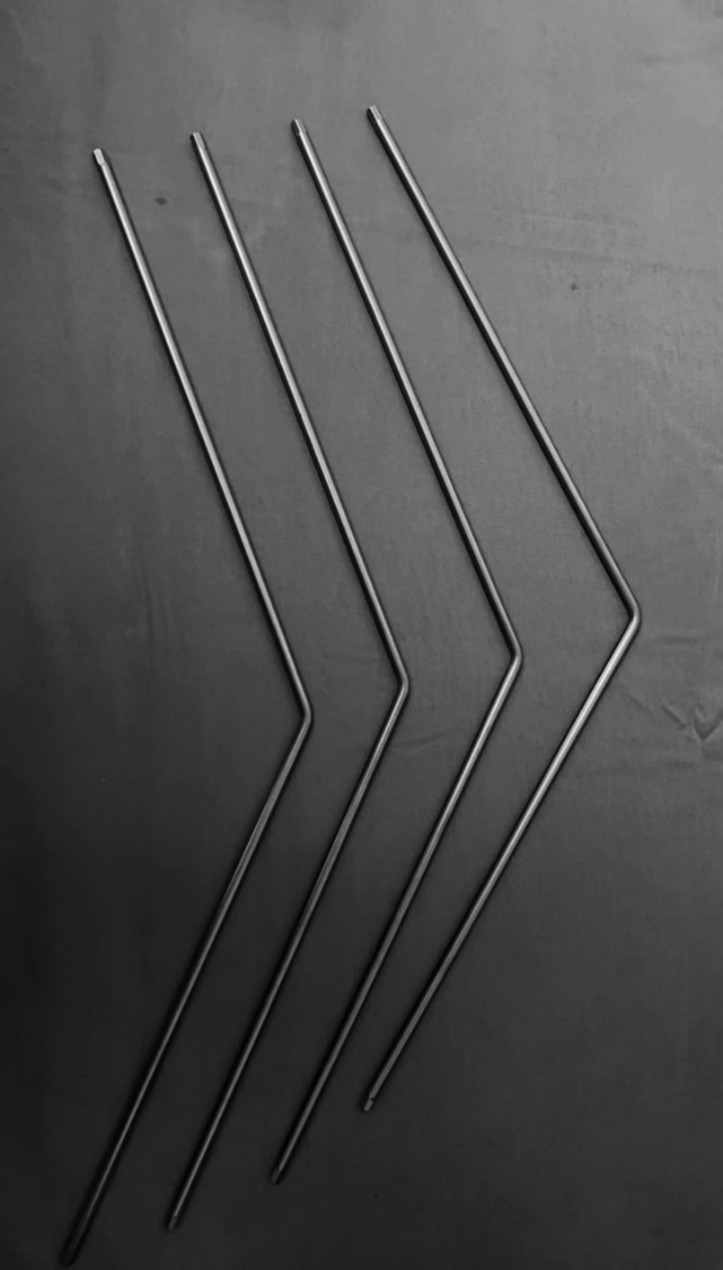
45° and 55° PV rods used in each case

Kyphotic correction was performed. The L3 vertebra was targeted, aiming for a PSO angle of 35–40° through a unilateral correction maneuver. First, a 45° PV titanium rod (Fig. 3) was bent accordingly and fixed to the caudal segment, with the corner of the PV rod located below the expected osteotomy site. Then, the extended screw holder was used to guide the reduction screws to the cranial portion of the PV rod, aiming to reduce the risk of coronal and sagittal translation during osteotomy closure.

Temporary rods were placed alternately to maintain the stability. Patient position was adjusted with the assistance of the enhanced positioning device and followed by cantilever and reduction maneuvers to restore lordosis. After the initial correction, the temporary rod was replaced with the other PV rod in the same manner. Compression was performed bilaterally and neurological monitoring through TCeMEPs and SSEPs was performed throughout to ensure protection of the spinal cord and nerves.

After correction, the wound was irrigated with saline and the lamina cortex of the surgical segments was removed. The excised bone was paved onto the fixed segments to facilitate spinal fusion. Two CoCr alloyed satellite rods were attached to the PV rods [13]. A drainage tube was placed in the operative wound and the surgical incision was sutured. For three months after the surgery, the patient wore the personalized thoracolumbar brace during daily activity.

## Results

Five male patients underwent one-level PSO with PV rod fixation. On average, the operation lasted 306.0 min, estimated blood loss was 765.0 mL, total autologous blood transfusion was 325.0 mL, and total allogeneic blood transfusion was 320.0 mL. One surgical complication occurred: an incidental durotomy resulting in a cerebrospinal fluid leak during osteotomy (Table 1). No other instrument-related or neurological complications occurred.

On average, general kyphosis (GK) was corrected from 68.2° to 22.0°, lumbar lordosis (LL) was restored from −5.4° to 37.6°, SVA was corrected from 231.8 mm to 89.0 mm, and CBVA was reduced from 42.6° to 13.8°. The correction angle ranged from 39° to 56° and the osteotomy vertebrae angle (OVA) was performed from 34° to 51° based on surgical necessity, with an average of 40.8° (Table 2).

**Table 1:** Surgical parameters of all 5 patients

No.	Gender	Age (yrs.)	Instrumented Segments	Osteotomy Type	Osteotomy Level	Duration (min)	Estimated blood loss (mL)	Blood Transfusion (ml)		Complications
								Autologous	Allogeneic	
1	M	44	T12-L5	PSO	L3	330	950	400	400	Dural tear and cerebrospinal fluid leakage
2	M	43	T11-L5	PSO	L3	275	1225	500	400	N
3	M	33	T12-L5	PSO	L3	315	550	225	400	N
4	M	47	T12-L5	PSO	L3	300	500	200	400	N
5	M	38	T12-L5	PSO	L3	310	600	300	0	N

**Table 2:** Radiological parameters of all 5 patients

Parameter	Preoperative	Postoperative	Improvement
GK	68.20 ± 16.99	22.00 ± 11.53	46.20 ± 6.46
TK	38.20 ± 16.66	39.40 ± 16.83	1.20 ± 0.84
TLK	18.00 ± 6.63	18.40 ± 7.77	3.20 ± 3.19
LL	-5.40 ± 6.80	37.60 ± 4.72	43.00 ± 7.38
PI	49.20 ± 13.18	46.60 ± 8.56	6.60 ± 2.97
PT	35.40 ± 5.94	25.20 ± 7.26	10.60 ± 8.65
SS	14.20 ± 9.18	21.60 ± 7.23	11.40 ± 6.47
SVA	231.80 ± 85.84	89.00 ± 65.46	136.40 ± 54.19
CBVA	42.60 ± 22.83	13.80 ± 7.66	33.00 ± 23.73
OVA	-4.00 ± 8.34	36.80 ± 5.22	40.80 ± 6.76

All data presented as Mean ± SD. All angular parameters measured in degrees; SVA measured in millimeters. Abbreviations: GK, global kyphosis; TK, thoracic kyphosis; TLK, thoracolumbar kyphosis; LL, lumbar lordosis; PI, pelvic incidence; PT, pelvic tilt; SS, sacral slope; SVA, sagittal vertical axis; CBVA, chin-brow vertical angle; OVA, osteotomized vertebra angle

## Representative case report (Patient #3)

A 33-year-old male patient with a 20-year history of AS and a 10-year history of progressive spinal kyphosis presented to our clinic. His kyphosis had recently begun to worsen, and he found it increasingly difficult to walk upright and lie flat on his back, symptoms which greatly hindered his ability to complete regular daily activities and worsened his mental health.

The patient was admitted to our hospital for surgery. Physical examination confirmed the kyphotic spine deformity and indicated poor spinal motion. No loss of muscle strength or sensory function was noted. Radiological examination showed a 95° GK deformity with 14° lumbar kyphosis and severe sagittal imbalance. MRI scans showed malformation of the spinal column, without lesion. CT scans revealed extensive spinal fusion from thoracic to sacral vertebrae.

A posterior one-level PSO at L3 using PV rods, alongside fixation and fusion from T12-L5 vertebrae, was planned and performed. Postoperative spinal X-ray showed that GK was corrected from 95° to 38°, the intended OVA of 51° was achieved, LL was restored from −14° to 42°, and SVA was corrected from 325 mm to 126 mm. Most importantly, CBVA was corrected from 59° to 19°.

**Fig. 4:**
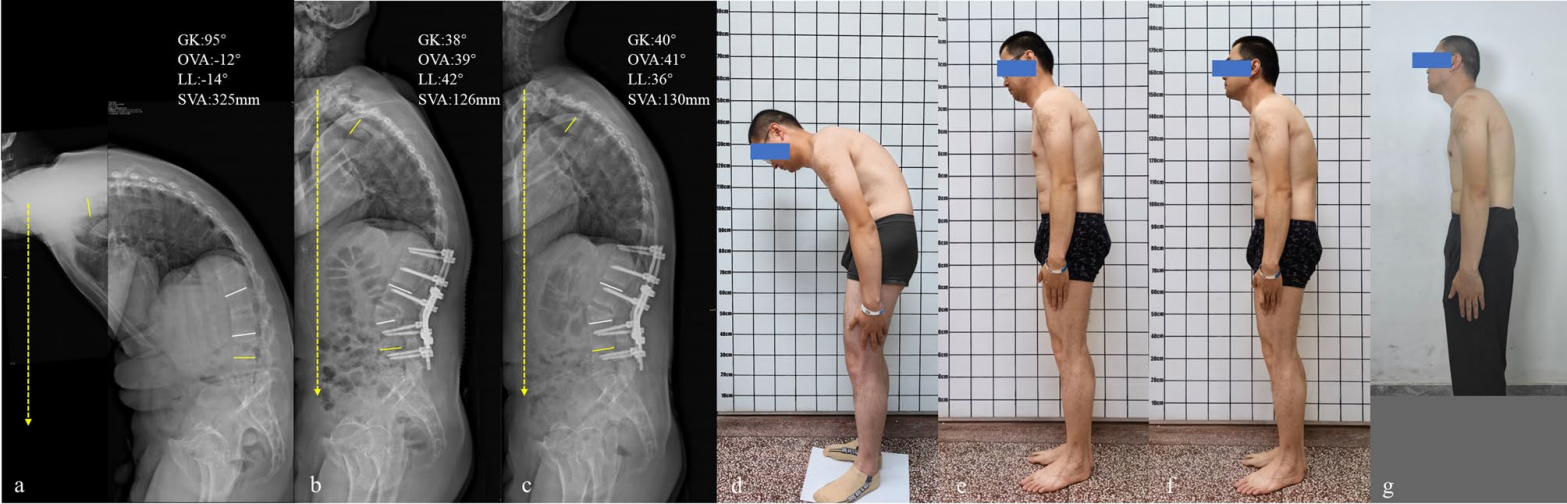
Patient #3: 33-year-old male patient with progressive spinal kyphosis secondary to AS. (**a**) preoperative standing lateral radiograph demonstrated severe thoracolumbar kyphosis with GK of 95° and SVA of 325 mm; (**b**) posterior one-level extended PSO at L3 combined with PV rod fixation and fusion from T12-L5, resulting in a 51° OVA correction at L3, with GK of 38° and SVA of 126 mm; (**c**) at 3-month follow-up, satisfactory spinal alignment was maintained, with GK of 40° and SVA of 130 mm; (**d**-**f**) clinical photographs illustrating changes in appearance, preoperatively (**d**), postoperatively with bowed head (**e**), postoperatively while maintaining horizontal gaze (**f**), standing at final postoperative follow-up (**g**)

After surgery, the patient was able to walk upright, maintain horizontal gaze, and lie flat. At a three-month follow-up, all radiological parameters were well-maintained. No iatrogenic complications were noted and the patient continued to enjoy a greatly improved quality of life (Fig. 4).

.

## Discussion

Ankylosing spondylitis (AS) is a disease commonly diagnosed in young males and characterized by progressive thoracolumbar kyphosis [14]. AS causes the spine to experience forward flexion, leading to severe kyphosis and a forward shift in the trunk’s center of mass. As such, AS patients often report difficulty standing upright and inability to maintain horizontal gaze [15]. Furthermore, to compensate for advancing kyphosis, patient posture adapts, pelvic pronation occurs, PT increases, and SS decreases, altogether resulting in an increased load on the trunk and lower limbs [16].

Existing literature has shown that spinal parameters such as CBVA, SVA, GK, LL, PI, PT, and SS are closely associated with quality of life for AS patients with kyphosis [17, 18]. As follows, the main goal of corrective surgery for such patients is to minimize the kyphotic deformity and restore sagittal alignment, often measured by a satisfactory postoperative CBVA that corresponds to improved physical function and appearance. Among many existing surgical options for AS-induced kyphosis, one-level PSO remains the gold standard. Another commonly used procedure, two-level PSO, is often used to treat severe cases of kyphosis, as it allows for greater sagittal parameter correction and a more optimal stress distribution in the implanted hardware. However, two- or multi-level PSO for one-stage surgery has been shown to lengthen operating time and increase patient blood loss, thus exacerbating the risk of surgical complications [6, 19].

Several modified one-level PSOs have been explored for the treatment of severe kyphosis, but these techniques have been largely focused on the treatment of traumatic thoracolumbar kyphosis rather than AS-induced deformities [20]. One technique, namely vertebral column decancellation (VCD), was previously established by Wang and Lenke to manage rigid thoracolumbar kyphotic deformities secondary to AS via controlled anterior column opening and posterior column closing without sagittal translation [21, 22]. This method was found to be a viable surgical option, but remains a technically demanding procedure which may present some of the same drawbacks seen in two- or multi-level PSO: prolonged operation time, increased intraoperative blood loss, and risk of surgical complication. As such, there remains a need for viable surgical options that treat AS-induced kyphosis without increasing the rate of complications. Thus, our group aimed to develop a novel technique which innovates upon a standard one-level PSO by adding PV rods at middle lumbar spine, aiming to correct kyphotic deformities without exacerbating surgical difficulty and complications.

Overall, our one-level PSO with PV rods achieved comparable or improved surgical outcomes in comparison to previous studies, including two prior works investigating the technically demanding VCD method. Herein, the mean GK correction was 46.2°, which exceeds that of previously reported results from one-level PSO (37.65°±9.16°, 26.7° ± 16.8°) and is comparable to that of one-level VCD (49.13°±9.87°, 38.3° ± 10.2°) [22, 23]. Regarding sagittal restoration, the average 136.4 mm correction yielded by our enhanced PSO was greater than that of one-level PSO (112.57 ± 29.36 mm) and VCD (110.83 ± 35.68 mm) reported by one prior study [22]. As for lumbar lordosis, the mean correction of 43.0° obtained herein exceeded that of one-level PSO reported by both prior works (33.69 ± 24.08°, 26.0° ± 21.7°) and was comparable to those of the VCD groups (44.93 ± 11.73°, 52.5° ± 16.2°) [22, 23]. Finally, the average 40.8° OVA correction obtained in this study was greater than the average 38.5°±6.1° correction achieved by one PSO group, but was less than that of VCD (50.8° ± 9.7°) [23]. Nevertheless, we believe that the lower LL and OVA corrections in our study can be attributed to our cohort’s high degree of preoperative kyphosis, to which the pre-planned osteotomy angle was adjusted accordingly. In addition, high degrees of correction (maximum 56° LL and 51° OVA) are achievable by one-level PSO with PV rods. We hypothesize that a study with a larger sample size and less preoperative kyphosis would yield LL and OVA corrections more comparable to that of VCD.

Considering the high rate of complications after PSO, our group made further innovations in order to minimize the risks of dura buckling, rod fatigue, and sagittal translation. Following the recommendations of existing literature, we enlarged the osteotomy area of the posterior column to include the partial removal of adjacent segments, aiming to minimize the risk of dura buckling [24]. Next, a rod angle of 45° was required for our patients. However, it is challenging to bend fixation rods to such sharp curves using traditional rod benders, and repeated attempts at notching and contouring rods has been thought to decrease rod strength, thus heightening the risk of complications [25]. In light of this issue, we utilized notch-free, pre-contoured V-shaped rods, giving surgeons greater control over the osteotomy angle while minimizing threats to rod fixation strength. The idea originated from personalized pre-contoured rod used in adult deformities. Finally, by implanting a PEEK cage, we aimed to maintain the height of the anterior column during surgery in order to prevent sagittal translation [26].

Next, our usage of an enhanced positioning device aimed to reduce intraoperative stresses that contribute to several major complications encountered by AS patients treated by PSO, including screw loosening, instrument failure, sagittal translation, and neurological deficits. During traditional PSO, the cantilever method and other kyphosis-correcting maneuvers exert significant force on the implants and spine, increasing the risk of screw loosening and instrument failure [22, 27, 28]. Prior studies have reported that 27–40% of patients who underwent open-wedge osteotomy for kyphotic correction experienced sagittal translation, and 15% of said patients reported neurological deficits [29]. Thus, we implemented an extended screw holder and enhanced positioning device to allow the rods to be gradually fixed to each screw, reducing local stress, coronal and sagittal translation during osteotomy closure. Furthermore, cranial and caudal postural reduction was monitored closely and performed cautiously via the enhanced positioning device, decreasing both the difficulty of correction and the risk of stress-related complications. Altogether, these techniques resulted in no nerve deficit, screw loosening, or instrument failure in our cohort. While CSF leakage was experienced by one patient, this event was likely not related to the deformity correction and the patient had no further complications occurred [28]. Finally, although the main focus of this study was one-level PSO for the treatment of AS-induced kyphosis, we believe that these surgical devices and approaches can be implemented into osteotomy procedures for the treatment of other spinal conditions.

This study presents several limitations. First, this is a retrospective study with a small sample size (*n* = 5), thus limiting the generalizability of its findings. Second, a control group undergoing traditional PSO was not recruited. Finally, all five patients presented with moderate to severe kyphosis, and some patients with severe preoperative deformity (large CBVA) accepted a limited correction of sagittal alignment in order to maintain horizontal gaze, which resulted in less correction than originally planned. This technical feasibility study focused on a homogeneous male cohort with severe deformities. Future multi-center studies including female patients and milder kyphosis are needed to establish broader applicability. What’s more, while early outcomes are encouraging, the absence of ≥ 2-year follow-up data limits assessment of long-term correction maintenance and late complications (e.g., rod fatigue, pseudarthrosis). Prospective studies with extended follow-up are underway. In the future, a randomized, controlled study with a large sample size and control group should be conducted in order to further verify the feasibility, safety, and capacity of this novel surgical technique. The biomechanical experiment of PV rod is currently underway.

## Conclusion

One-level PSO with pre-contoured V-shaped rod fixation at middle lumbar spine is a feasible alternative to existing surgical options for kyphotic correction and lumbar lordosis restoration in AS patients. This technique obtained satisfactory radiographic and clinical outcomes for patients with rigid lumbar kyphosis without requiring additional surgical procedures or introducing further risk of complications.

## Data Availability

The data has been provided in the manuscript. Please contact the author for more additional data.
